# Forensic reconstruction of two military combat related shooting incidents using an anatomically correct synthetic skull with a surrogate skin/soft tissue layer

**DOI:** 10.1007/s00414-018-1802-z

**Published:** 2018-03-07

**Authors:** Peter Mahoney, Debra Carr, Karl Harrison, Ruth McGuire, Alan Hepper, Daniel Flynn, Russ J. Delaney, Iain Gibb

**Affiliations:** 1 Cranfield Forensic Institute, Cranfield University at the Defence Academy of the United Kingdom, Shrivenham, Swindon, SN6 8LA UK; 2 Royal Centre for Defence Medicine, ICT Centre, Research Park, Birmingham, B15 2SQ UK; 3 Centre for Defence Engineering, Cranfield University at the Defence Academy of the United Kingdom, Shrivenham, Swindon, SN6 8LA UK; 4 Present Address: Defence and Security Accelerator, Porton Down, Salisbury, Wiltshire, SP4 OJQ UK; 5 Defence Science and Technology Laboratory, Porton Down, Salisbury, Wiltshire, SP4 0JQ UK; 6 Cranfield Ordnance Testing and Evaluation Centre (COTEC), Cranfield University, West Lavington, Devizes, Wiltshire, SN10 4NA UK; 7 South West Forensic Pathology Group Practice, Box 388, Bristol, PO BS9 0DB UK; 8 X-ray Department, Medical Centre, HMS Nelson, HM Naval Base Portsmouth, Hampshire, PO1 3HH UK

**Keywords:** Ballistic, Helmet, Human head surrogate, Cranial trauma, Simulation

## Abstract

Six synthetic head models wearing ballistic protective helmets were used to recreate two military combat-related shooting incidents (three per incident, designated ‘Incident 1’ and ‘Incident 2’). Data on the events including engagement distances, weapon and ammunition types was collated by the Defence Science and Technology Laboratory. The models were shot with 7.62 × 39 mm ammunition downloaded to mean impact velocities of 581 m/s (SD 3.5 m/s) and 418 m/s (SD 8 m/s), respectively, to simulate the engagement distances. The damage to the models was assessed using CT imaging and dissection by a forensic pathologist experienced in reviewing military gunshot wounds. The helmets were examined by an MoD engineer experienced in ballistic incident analysis. Damage to the helmets was consistent with that seen in real incidents. Fracture patterns and CT imaging on two of the models for Incident 1 (a frontal impact) were congruent with the actual incident being modelled. The results for Incident 2 (a temporoparietal impact) produced realistic simulations of tangential gunshot injury but were less representative of the scenario being modelled. Other aspects of the wounds produced also exhibited differences. Further work is ongoing to develop the models for greater ballistic injury fidelity.

## Introduction

Reconstructions are used in forensic investigation to try and understand what happened during an incident. Shooting incident reconstructions can vary in complexity from a single shot being fired from a single weapon to multiple weapons firing many shots [1]. Reconstructions can range from scale models and computer animations through to full-sized re-enactments [1]. The aim of this project was to attempt to reconstruct two examples of combat-related ballistic head injury.

Gunshot injury in humans can take a multitude of forms as detailed by Di Maio [2] and vary according to weapons system used, bullet construction and area of the body impacted. These factors need to be considered when contriving a reconstruction, particularly as different ammunition types can produce different bone fracture patterns [3] and injuries [4].

Reconstructions of ballistic events on humans have been undertaken with a range of models as described in Humphrey and Kumaratilake’s recent (2016) review [5]; this includes cadavers, animal models, simulated bone and tissue and computer models. Raino et al. [6] used anaesthetised pigs to study the morphology of assault rifle gunshot wounds and subsequent post-mortem changes as part of their investigation into shootings in Kosovo. They commented that the work was helpful for clarifying injury mechanism but that ‘*the reproducibility of ballistic experiments using live animals is extremely difficult’* [6]*.* Issues included both variability in the appearance of different wounds, despite being inflicted by the same weapons and ammunition, and the effect of post-mortem changes on the wounds as the experiment progressed.

Smith et al. [7] assessed Synbone®, a polyurethane synthetic bone substitute, against real bone in a series of ballistic experiments. The advantage of using a manufactured proxy is that each should be identical. They concluded that the Synbone® responded similarly to the bone on a macroscopic level but, unsurprisingly, was less comparable when examined in detail due to the structural differences between the materials. The Synbone® spheres shot with modern rifles (.243 in. and 7.62 mm calibres) were noted to ‘*compare favourably with published examples of modern cranial gunshot inju*ry’ [7]. Carr et al. [8] reported similar findings using an anatomically correct skull model which has been the basis for our subsequent experiments [9, 10].

Much of the groundwork on simulating ballistic head injury has been done by Thali et al. [11] who developed a ‘Skin-skull-brain model’ made of a silicone scalp, a layered polyurethane sphere to represent the skull, and gelatine 10% at 4 °C to simulate brain. After shooting the model with a series of ammunition types, the authors reported that the results were comparable to those of real gunshot injuries. Thali et al. [12] went on to use their model in a series of experiments, including researching the behaviour of ‘glancing’ head gunshots. They concluded that the model could be used for answering questions in real forensic cases where this was the underlying injury mechanism, i.e., it would provide a faithful platform for reconstructions in casework.

In more recent work, Synbone® polyurethane spheres have been used to model close range (30 cm) ‘execution’ style head gunshots. The authors used six different calibres and provide photographs of two clinical cases (.22 LR and .45 ACP) where the bony injury and the model look very similar [13]. The model also performed well in a reconstruction of a blunt impact on a Neolithic skull using a replica contemporary club [14].

In modern combat injury, the effect of protective helmets needs to be considered when modelling ballistic wounds [15]. In our previous work [16], the addition of material layers (including simulated bone, skin and sheets of helmet material) in front of a 10% gelatine block tended to increase the variability in bullet behaviour between different shots. This suggests that reconstructing a bullet impact on a head wearing a helmet is likely to be more complex than one without. Impact with intermediate targets (such as bone or helmet material) may also cause bullets to destabilise and fragment [17], adding further to the complexity. Impacts on clothing [18] can also influence bullet stability.

It is not possible to place a combat helmet on a Synbone® sphere. In order to reconstruct impacts on a head wearing a modern combat helmet, we have been developing a surrogate around an anatomically correct polyurethane skull [8] which, under ballistic impact, produces realistic fracture patterns [9]. Differences in bone thickness and structure within the skull accounting for fracture patterns from contact gunshot wounds are discussed by Fenton et al. [19] which lends further weight to using anatomically correct models for complex reconstructions.

Studies investigating Behind Helmet Blunt Trauma (BHBT) have looked at the interaction between ballistic impact, protective materials and head injury. Sarron et al. [20] undertook two sets of experiments using initially dry skulls, and later cadaveric heads, both protected by plates of helmet materials. The models were also instrumented with pressure sensors. The helmet materials were placed 12 to 15 mm from the skulls and impacted with 9-mm bullets at around 400 m/s. The aim was to produce a non-penetrating impact on the plates and assess the damage to the skulls and cadaveric heads from the plate deformation. For the cadaveric heads, a 4-point scale was used to assess damage (0, nothing, to 3, severe). Greater plate deformation and plates placed closer to the models were associated with more damage to the models.

Freitas et al. developed a ‘Human Head Surrogate’ [21] (HHS) by combining human crania with synthetic soft tissues and brain mounted to a Hybrid III (‘crash test dummy’) neck assembly. A stated intent was to *fill the void betweenpost-mortemhuman subject testing* (*which have biofidelity but are subject to handling restrictions) and commercial ballistic head forms (easy to use but lack biofidelity)* [21]. The models were instrumented with pressure transducers. The surrogates were fitted with a protective helmet and impacted with a series of ammunition types. As the intent of the study was to look at BHBT and, as with Sarron et al. [20], produce a non-penetrating impact, a ceramic applique was fitted to the front of the helmet for high energy ammunition (7.62 × 39 and 7.62 × 51 mm). Flash X-ray was used to capture the maximum back face deformation of the helmets. The extent of the resulting fractures was assessed and graded as none, minor, moderate or critical, descriptors which had been discussed earlier in the study in relation to associated clinical injury.

In contrast to these BHBT studies, we wanted to assess a completely synthetic surrogate and test it against a penetrating head injury.

## Method

Ethical approval for developing and testing a ballistic injury surrogate was obtained from Cranfield University.

Permission to view anonymised Computer Tomography (CT) images of deceased coalition service personnel was granted by the Coroners of Oxford and Wiltshire. The request to the coroners stated that the purpose of this was to develop a synthetic model of ballistic head injury to improve future protection.

The Joint Theatre Trauma Registry (JTTR) is a data base of major trauma casualties from recent conflicts [22]. Permission was granted to search JTTR for fatal gunshot head injuries, building on previous work [23]. The review of JTTR took place at the Defence Science and Technology Laboratory (DSTL) and identified 60 casualties who had suffered a fatal gunshot wound to the head in the period between 2006 and 2013.

Each case was then assessed for additional information about the events using incident reporting, contemporary accounts, equipment and threat analyses, and operational learning reports held at DSTL. This included likely engagement ranges, weapon systems used and bullet types (where known). If engagement ranges were not specifically stated, these were calculated from maps and satellite images of the ground where the shooting took place.

All reviews were conducted using incident reference numbers, and no personal data was released in accordance with data protection requirements.

In order to allow comparison with our previous work [9, 10, 16], casualties were identified where 7.62-mm bullets were confirmed responsible for the injuries (this included 7.62 × 39, 7.62 × 51 and 7.62 × 54R mm). Seven casualties were confirmed as such. While it is highly likely that other casualties were struck by 7.62-mm bullets, this could only be confirmed with certainty where either bullets or enemy weapon systems were recovered.

**Fig. 1:**
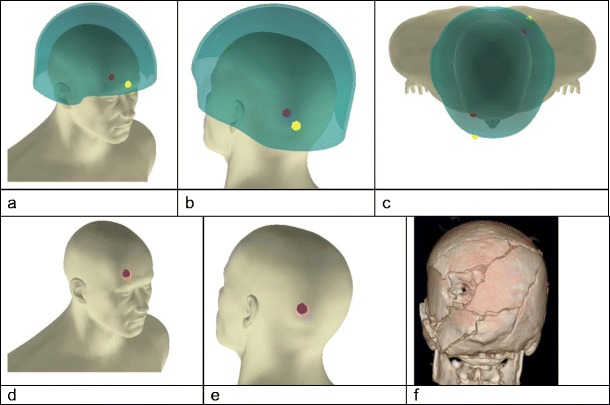
IMAP images from Incident 1. **a** Entry wounds, helmet in situ. **b** Exit wounds, helmet in situ. **c** View from above, helmet in situ. **d** Entry wound site on skin. **e** Exit wound site on skin. **f** Anonymised CT scan showing exit wound left parietal bone, posterior aspect

DSTL uses a software package called IMAP (Interactive Mapping Analysis Platform, IMAP v1.3.3.0, developed under contract to DSTL) to map bullet and fragment strikes, trajectories and resulting injuries on casualties. The IMAP images for the seven casualties with 7.62mm bullet injuries were reviewed and confirmed to involve the casualties’ helmet and head (Fig. 1a–e).

Casualties where bullets had hit an intermediate target prior to striking the helmet were removed from the group. As noted above, this makes bullet behaviour more unpredictable. Casualties whose injury predated the UK policy of post-mortem CT scanning were also removed from the group. This left two casualties, identified only by an incident number. The incident number was used to retrieve the post-mortem CT scans, and selected images from these were made by Defence Radiology with all identifying data removed (Fig. 1f).

The anonymised CT images and summary of the incidents (engagement range, calculated impact velocity of the bullet and selected IMAP images) were collated as laminated A4 sheets, one set for each shooting, and labelled ‘Incident 1’ and ‘Incident 2’.

**Fig. 2:**
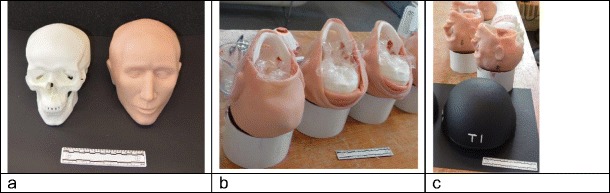
Model construction. **a** Polyurethane skull and corresponding skin layer. **b** Models being filled with 10% gelatine. **c** ballistic helmet in foreground

Six head models were built from a synthetic skull [8–10], face and scalp. The data for the skulls comes from 3-D mapping of a human post-mortem specimen in Tai Wan. The skulls are made from a two-part thermoset polyurethane plastic mixed together within a vacuum casting chamber (Craig Vickers, ARRK Europe Ltd., Gloucester Technical Centre, Olympus Park, Quedgeley, Gloucester, Gloucestershire GL2 4NF personal communication, 2016). The skulls are produced in two parts (above and below the post-mortem cut line) which allows the bone thickness and internal structure of the skull to be reproduced accurately but means that the parts need to be bonded prior to ballistic tests. The skin and soft tissue was made using polydimethylsiloxane, PDMS (Flexural Composite Research Laboratory, Nottingham Trent University, Nottingham, NG1 4GG). The ‘brain’ is made with 10% (by mass) gelatine (Fig. 2a, b). The full methodology is described in [10].

Each head model was fitted with a commercially purchased ballistic helmet (Fig. 2c). The helmet consisted of an outer protective shell made of multiple layers of resin-bonded para-aramid and an impact absorbing liner. For security reasons, this was not a current in-service military helmet but one with a similar construction and performance to allow a valid comparison.

The models were placed in turn 9.6 m from a No 3 Enfield Proof Mount fitted with an accurate barrel (length 72.5 cm, 1:9.45 twist rate) at the Cranfield Ordnance Testing and Evaluation Centre (COTEC, Gore Cross, West Lavington, Devizes, Wiltshire, SN10 4NA, UK).

**Fig. 3:**
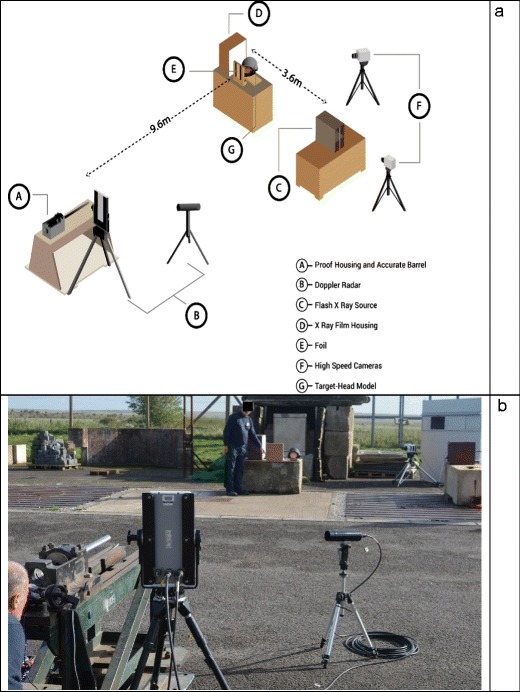
Experimental setup. **a** Schematic. **b** Range at COTEC

Each model was shot once with 7.62 × 39mm Mild Steel Core Ukrainian Ammunition [9, Fig. 1. 10, Fig. 3]. Using data from Kneubuehl [24] and data from previous work at the Impact and Armour Group [25], ammunition was reloaded with Vivhtavuori N140 smokeless propellant (Nammo Lapua Oy, Vivhtavuori Site, Ruutitehaantie 80, FI-41330 Vihtavuori, Finland) to recreate the bullet impact velocities from the actual incidents. Models 1 to 3 were used to recreate Incident 1 (entry and exit wounds as shown in Fig. 1), and Models 4 to 6 to recreate Incident 2 (entry wound left temporoparietal region; exit wound lower left occiput). Models for Incident 1 were impacted at a mean velocity of 581 m/s (SD 3.5 m/s), and models for Incident 2 at a mean velocity of 418 m/s (SD 8 m/s).

The impacts were captured with high-speed video (HSV) cameras (V12; sample rate 41,000 fps, exposure 10 μS, resolution 512 × 256; and V1212; sample rate 37,000 fps, exposure 6 μS, resolution 384 × 288). Just prior to impact, each bullet penetrated a thin foil located in front of the model triggering the Scandiflash 150 X-ray system (Scandiflash AB, Palmbladsgatan 1A, S-754 50 Uppsala, Sweden). The distance from the foil to the centre of the model was measured and, with the expected impact velocity of the bullet, used to calculate the likely time lapse in microseconds from the bullet cutting the foil to reaching the required point in the model, similar to the method described by Freitas [21]. This was input into the X-ray system’s delay generator with the aim of delivering the X-ray exposure at the correct point in the bullet’s pathway. Each exposure was delivered over a period of 35 ns. To ensure adequate penetration of the model, the maximum output voltage of 150 kV was used. The experimental setup is summarised in Fig. 3.

After shooting, each model was handled carefully to minimise any disruption to the underlying bullet damage and taken in padded cool boxes to the Department of Radiology at the Queen Elizabeth Hospital Birmingham for CT Scanning by military radiographers using a SOMATOM Definition CT scanner (Siemens Health Care Ltd., Camberley, UK) with Spiral Head protocols (Window Level 100/35, 1 mm slice thickness). The scans were sent to an experienced military radiologist for reporting and comparison with the actual incidents. Tissue and helmet layers were removed from the images using Phillips Brilliance Extended Work Station (Koninklijke Phillips N.V., Amstelplein 2, 1096 BC Amsterdam, The Netherlands).

**Fig. 4:**
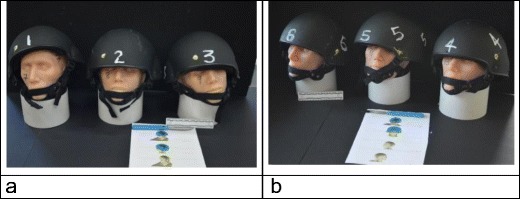
Models awaiting examination at the Defence Academy. **a** Incident 1, models 1–3, frontal impact. **b** Incident 2, models 4–6 side impact. Hard copy of the relevant IMAP images (described above) is visible in the foreground. Models are described in the text by the helmet number shown in white. The black numerals on the faces indicate that they are sequential to those described in [10]

The models were then taken back to the Impact and Armour Group at the Defence Academy, Shrivenham, for examination by a Home Office pathologist and an MoD engineer experienced in post-incident analysis of ballistic events (Fig. 4).

As shown in Fig. 4a, b and Fig. 9b, the helmets had all been moved by the bullet impact from the position in which they would be correctly worn on the head. The radiologist and pathologist were invited to score their findings using a Likert-type scale [26] (Table 1, ‘Results’) against the actual incidents being recreated (Table 2, ‘Results’). They were also asked to comment on how the models compared to other incidents they had been involved with. The MoD engineer was invited to write up the findings in the format that would be used in actual investigations.

**Table 1:** Likert-type scores

(4) Exactly like the real incident	(3) Quite like the real incident	(2) A bit like the real incident	(1) Nothing like the real incident

**Table 2:** Summary of Likert-type scores for Forensic Pathologist (FP) and Military Radiologist (MR). FP scores based on examining the models which did not include the helmet which was being assessed by the MoD Engineer or the CT scans; MR scores based on examining the CT scans

	Helmet CT imaging	CT Imaging of head model	Skin entry	Bone entry	Bullet path in brain	Bone exit	Skin exit	How close is the model taken as a whole to the incident?
Incident 1								
Model 1								
FP	–	–	2	2	3	3	2	2
MR	4	2	3	2	3	3	2	2
Model 2								
FP	–	–	2	2	3	3	2	3
MR	4	3	3	3	3	2	1	3
Model 3								
FP	–	–	2	2	3	3	1	3
MR	4	3	3	3	3	3	1	3
Incident 2								
Model 4								
FP	–	–	2	1 (3)^b^	1 (3)^b^	1(3)^b^	2	1 (3)^b^
MR	3	3	3	3	2	3	1	3
Model 5^a^								
FP	–	–	–	–	–	–	–	–
MR	4	–	–	–	–	–	–	Helmet damage only
Model 6								
FP	–	–	2	3	2 (3)^b^	3	3	2 (3)^b^
MR	4	4	3	3	3	4	2	(4)^b^ (excluding skin)

^a^Model 5 was helmet damage only

^b^Number in brackets indicates how well these models represent a tangential bullet strike; number outside brackets assesses the model against the actual incident (see text)

## Results

### Impact event HSV and flash X-ray.

**Fig. 5:**
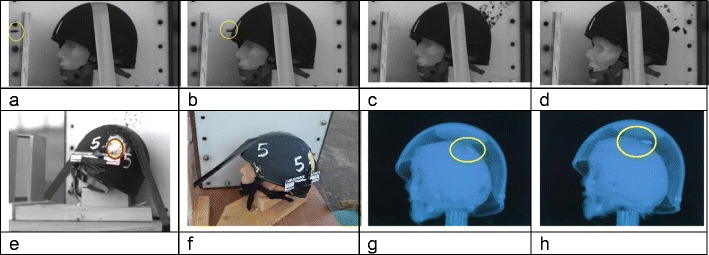
**a**–**d** Impact sequence model 1, frames from V12 camera. **a** Bullet (circled) prior to cutting foil. **b** Helmet impact, bullet circled. **c** Shower of paint from bullet impact inside rear of helmet. **d** Distortion of face due to stretching from temporary cavity development. **e** Impact of bullet inside rear of helmet 5. Distortion of helmet material from bullet impacting sideways circled. Frame from V1212 camera. **f** Model 5 in situ after shooting. **g**–**h** Flash X-ray images. **g** Model 3. **h** Model 2. Bullets circled. Forensic scale visible in panels **e** and **f**.

Impact events were captured on HSV (V12 and V1212) for all six models. Flash X-ray imaged the bullet passage in models 2, 3 and 5. Bullets perforated all the head models except 5 where the bullet passed between the inside of the helmet and the head, impacting on the inner aspect of the rear of the helmet. The forward facing surface of each helmet was perforated by the bullet entry impact (Fig. 5b). None of the rear surfaces of the helmet were perforated after the bullet exited the head model, although damage is visible on the CT scans (Fig. 9e).

Example images from the HSV and flash X-ray, plus model 5 in situ after shooting, are shown in Fig. 5. Of note, the bullets can be seen to have yawed through 180° in the flash X-ray images (Fig. 5 g, h).

### Summary of bullet trajectories

**Fig. 6:**
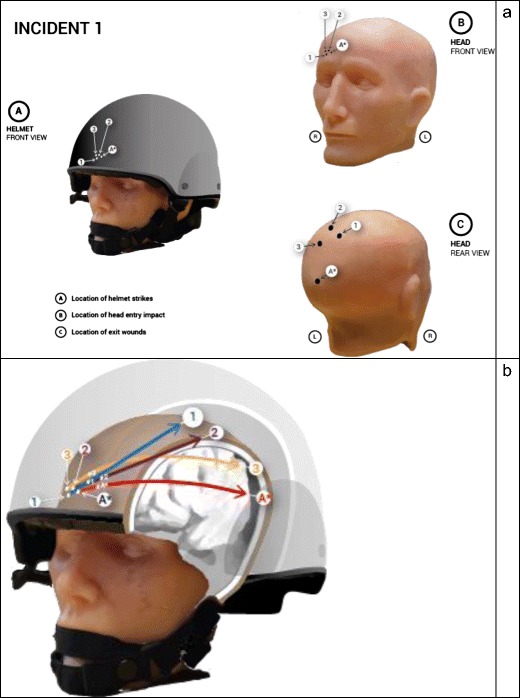
**a** Location of bullet strikes in models 1, 2 and 3. The A* symbol designates the actual strike points in Incident 1. **b** Summary of bullet trajectories within models 1, 2 and 3 compared with the actual trajectory, A*

**Fig. 7:**
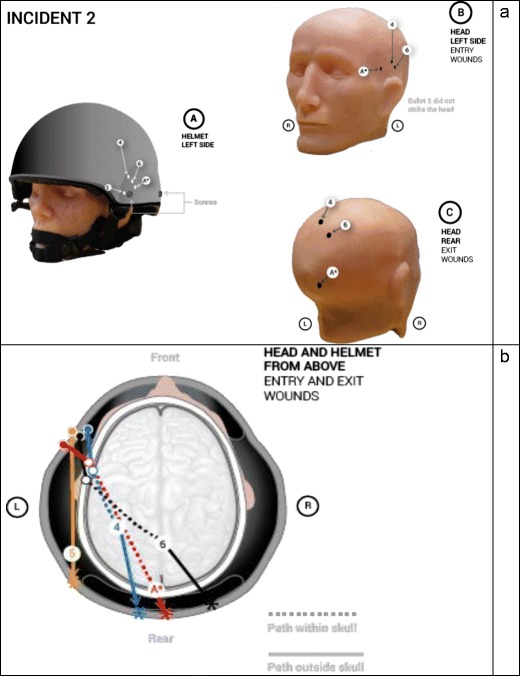
**a** Location of bullet strikes in models 4, 5 and 6. The A* symbol designates the actual strike points in Incident 2. **b** Summary of bullet trajectories within models 4, 5 and 6 compared with the actual trajectory, A*

The entry points in the helmets and entry and exit points in the head model were plotted to allow comparison with the actual incidents. These are summarised in Figs. 6 and 7. Of note, the experimental gunshots tended to track in a more upward direction than the actual wounds in both incidents.

### Engineering helmet assessment

**Fig. 8:**
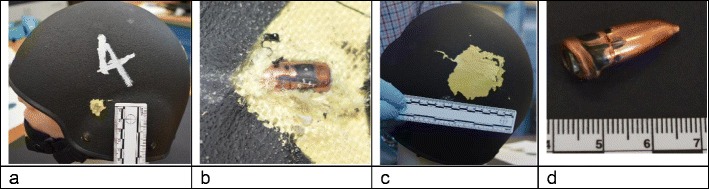
**a** Assessment of entry site, helmet 4; 4 mm perforating entry hole surrounded by area of paint loss. **b** Bullet embedded in composite shell of helmet 2. **c** Area of damage rear of helmet 1. **d** Distorted bullet from helmet 3. Copper jacket twisted to reveal mild steel core

All helmets had a perforating entry hole (all six were 4-mm diameter) (Figs. 5b and 8a), marked by bullet wipe, on the outer face of the helmet shell. The helmets were dismantled removing the inner net liner, foam impact liner and comfort pads to allow full inspection of the composite para-aramid shell. The inner face of the entry holes had fibres of the composite shell distorted inwards towards the head model. Helmets 1, 2, 3, 4 and 6 had fragments of simulated bone and tissue evident inside the helmet, consistent with a bullet passing through the head model.

A bullet was found lodged between the para-aramid shell and foam impact liner in helmets 1, 4 and 6. A bullet fell free from helmet 3 during examination (Fig. 8d). The bullets from helmet 2 and helmet 5 were embedded in the para-aramid shell (Fig. 8b).

On each of the helmets, there was an area of loss of the outer gel coat and black paint (Figs. 5c–f and 8c) (mean diameter 65 mm; SD 13 mm) with distortion of the para-aramid shell outwards (Figs. 5e and 8c).

Overall, the damage to the ballistic helmets was assessed to be representative of that seen in actual incidents.

### Forensic pathologist and military radiologist assessment

The Likert-type scores from the forensic pathologist (FP) and military radiologist (MR) are summarised in Table 2. As shown in the table, there were differences in the scores awarded by the pathologist and radiologist to the considered features.

**Fig. 9:**
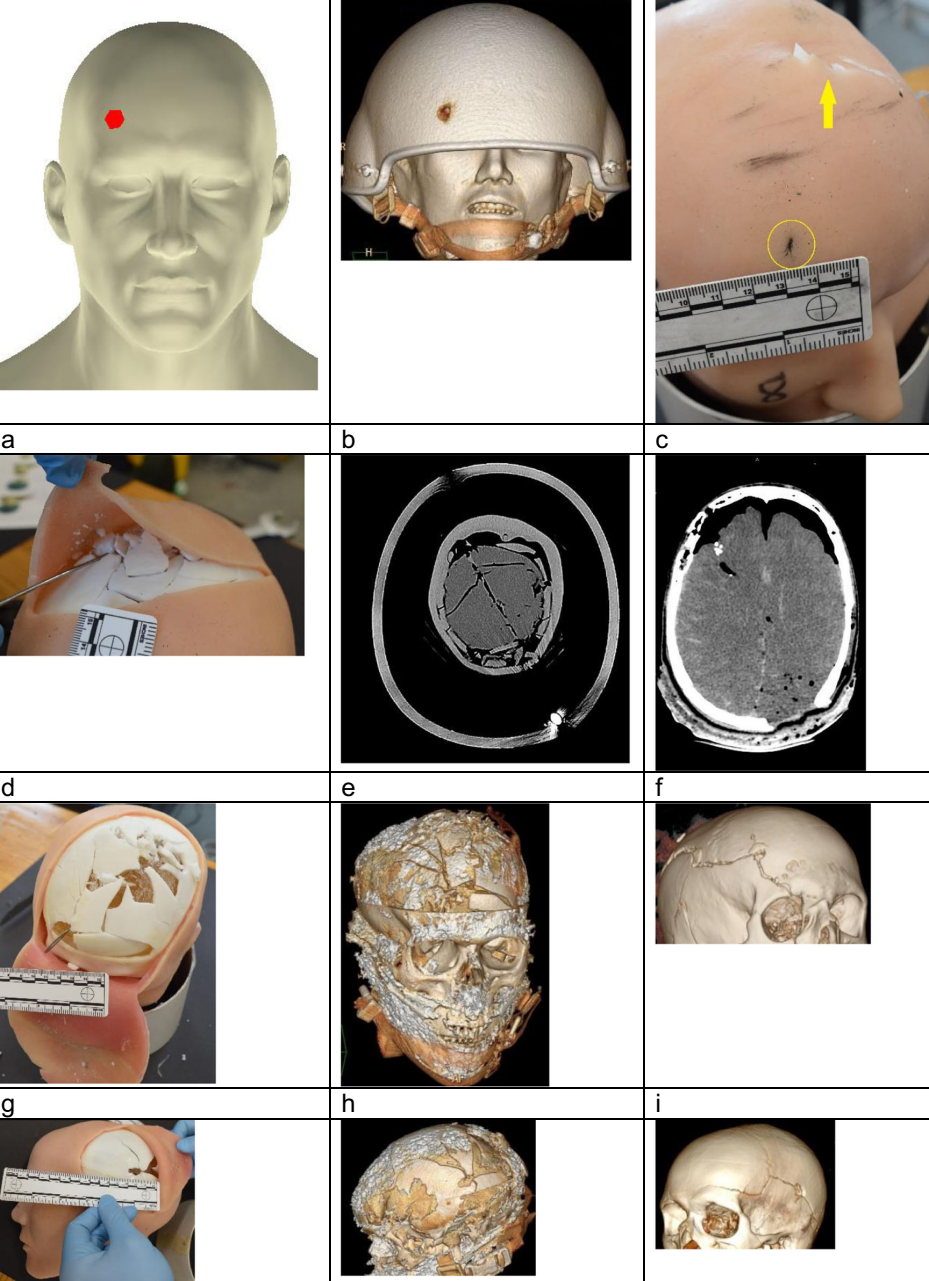
Examples of assessments of the simulated injuries and corresponding CT images; 3D CT reconstructions from actual incidents as detailed. **a** IMAP image for Incident 1. **b**–**e**, **g**–**h** Model 2, Incident 1. **b** 3D CT scan reconstruction with helmet in situ; entry site visible, for comparison with panel **a**. Note that helmet has been moved from correct wear position by bullet impact—see Fig. 4. **c** ‘Slit like’ skin entry wound (circled), and synthetic bone fragments protruding through the skin (arrow). **d** Underlying fractures. **e** Corresponding CT scan of the Model, helmet in situ. **f** CT scan of the actual incident, no helmet. **g** Model 2, fractures exposed, probe marks path of bullet through gelatine brain. **h** Corresponding 3D CT reconstruction. **i** 3D CT reconstruction of actual Incident 1. **j**–**k** Model 6, Incident 2. **j** tangential bullet strike; left parietal area. **k** corresponding 3D reconstruction of the fractures in model 6. **l** 3D CT reconstruction, actual incident 2

The CT imaging of the damaged helmets scored high, which is consistent with the engineering assessment given above. Model 5 only involved helmet damage so is not part of the further assessment of the simulated injuries. Examples of the simulated injury assessments and related CT images are shown in Fig. 9.

For Incident 1, models 2 and 3 scored ‘quite like the real incident’ for both the CT images and pathology assessments. The comment on the lower scoring model 1 was that the fracture patterns and fragments along the vertex did not seem quite right.

The skin entry wound was described a ‘slit like’ and too narrow in models 1–3 by the pathologist but ‘gaping’ by the radiologist. Synthetic bone fragments were noted to be protruding through the skin in models 2 and 3 in the area between the bullet entry and exit wounds.

For Incident 2, models 4 and 6 produced very superficial bullet paths compared to the actual incidents and were scored low by the pathologist accordingly. The imaging scores were generally higher. The models did, however, produce realistic tangential injury patterns of the type described by Thali [12] which is noted by the scores in brackets in Table 2. A key feature of model 6 was a good fracture propagation seen in the CT images.

Mean neck length (distance from entry into the gelatine to beginning of yaw) of the permanent cavity in the gelatine ‘brains’ of models 1–3 was 60 mm (SD 5 mm). The bullet path in the gelatine brains of models 4 and 6 was too small due to the tangential strikes to make meaningful measurements. The mean neck length in gelatine blocks with sheets of the same synthetic materials as intermediate targets described in [16] was 56 mm (SD 27 mm), but the SD was much greater.

## Discussion

The aim of this work was to attempt to replicate the injuries seen in two cases of combat-related ballistic head injury, building on our previous model development [8–10, 16].

Hueske [1] describes how shooting incident analysis and reconstruction requires the input of a number of different scientific disciplines. Our current work illustrates this when compared with our earlier projects by the input needed from DSTL to gather basic data about the events (engagement range, weapon type, ammunition type, etc.).

Two of the models representing Incident 1 achieved an overall score of 3 by the two assessors (‘quite like the real incident’), although as noted in the ‘Results’, the bullets in the models followed an upward path compared with those in the actual incident. Hueske [1] also notes that some variables about an incident will not be known including exact position of the shooter and the victim at the time. Small changes in the positioning of the models could alter the bullet path through the simulants significantly. In addition, as shown in our earlier work [10, Fig. 8], there is often a degree of variation in trajectories. From analysis of the HSV, the foil used to trigger the flash X-rays does not appear to alter the bullet flight prior to impact on the model. Bullet behaviour within the models could be inferred from the permanent cavity in the gelatine brain, the exit fracture patterns and the resting place of the bullet within the helmet structure, and the flash X-ray images were helpful to confirm this. While the mean neck length in the gelatine brains of models 1–3 (see ‘Results’) was similar to that of gelatine blocks in our earlier work [16] with intermediate targets of sheets of the same helmet material, synthetic skin and synthetic bone, there was greater variability in the blocks. Further work is needed to understand how comparable the models are.

The presence of synthetic bone and tissue within the helmets is consistent with post-shooting artefacts seen in actual incidents.

The models representing Incident 2 scored less well than Incident 1. The bullet pathway in synthetic head models 4 and 6 was very superficial. While these did not replicate the injuries from the actual incident, they did produce a good representation of tangential head gunshot wounds as described in Thali’s work [12] and illustrated in real examples by Di Maio [2].

Post hoc matching of damage to a model with historical clinical images is a useful process when establishing if a simulation has any clinical congruence [7–9, 11, 12], but when undertaking a reconstruction, caution and care are needed to ensure that incorrect conclusions are not drawn.

As shown in Fig. 7b, the bullet in model 5 perforated the helmet, missed the head and impacted in the rear of the helmet. The head model was undamaged. Within our military data set, there are at least two confirmed incidents of bullets entering helmets and missing the head. One bullet was retained in the helmet, one exited. In neither case was the skin penetrated, but both cases were associated with a traumatic subarachnoid haemorrhage and one with a calvarial fracture.

**Fig. 10:**
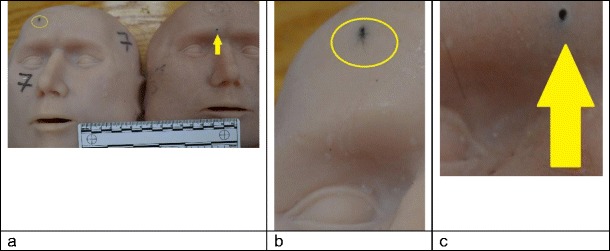
**a** Left hand ‘face’ model 1, this study; slit like entry circled; right hand ‘face’ model 3, study [10]; shot without helmet; circular entry arrowed; forensic scale visible. **b** Close-up entry wound from left hand face in panel **a**, this study. **c** Close-up entry wound, study [10], right hand face from panel **a**

There are acknowledged limitations to our model. One of these is the post-mortem cut line in the skull, discussed in [9, 10], which interferes with fracture propagation. Another is the extendable nature of the synthetic skin, discussed in [10]. Work is ongoing to address the skin properties, but we elected to shoot these models with the known skin material to assess if the presence of the helmet altered the skin wounding appearances. The skin entry wounds were described as slit like by the pathologist (Fig. 10a) but could be stretched to resemble the round entry wounds seen in models shot in our earlier work without helmets [10], (Fig. 10c).

**Fig. 11:**
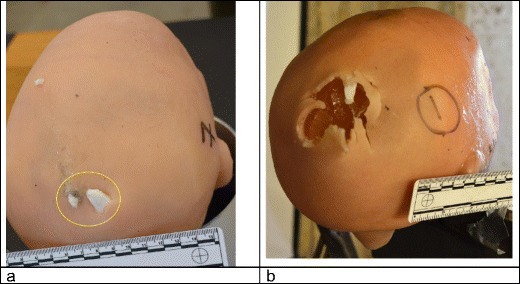
Exit wounds, rear of head models. **a** Model 1, this study, shot with helmet in situ; example exit wound with synthetic bone fragments protruding (circled). **b** Model 1 from study in reference [10]; shot without helmet. Exit wound is gaping with ragged edges

One explanation is that the synthetic skin underwent a degree of compression with the helmet in situ. The skin exit wounds scored lower in models 1–4 when compared with the models shot without helmets [10]. The wounds in the models without helmets tended to be larger with a more ‘ragged’ appearance [10] (Fig. 11). Observations from one of our authors (RD, forensic pathologist) is that the exit wound appearances in real casualties are variable, but with helmets in place, there may be less stellate type tearing than expected, presumably due to the support provided by the helmet to the tissues.

A question that needs further consideration is to what extent anatomically accurate models are needed for ballistic experiments or whether simple spheres suffice (other than the need for anatomical accuracy when placing helmets onto surrogates). Synbone® spheres have been successfully used for a variety of impact scenarios both ballistic [11–13] and blunt [14]. The internal structure of the skull does, however, influence the fractures that develop with ballistic impact as described by Fenton et al. [19], an effect that is seen in our skull model when fracture lines run through the skull base. How our models compare with the more biofidelic surrogates of Sarron et al. [20] and Freitas et al. [21] is a subject for future work.

## Conclusions

Six surrogate head forms were shot with 7.62 × 39 mm ammunition in an experiment to reconstruct two military shooting incidents of individuals wearing ballistic protective helmets (three models used per incident). Both sets of models exhibited a range of bullet trajectories despite factors such as bullet manufacturer, batch and propellant load being controlled.

The wounds, fracture patterns and CT images were compared with those from the actual incidents.

Two of the models used for Incident 1, a frontal impact, produced injuries closer to the actual event than did the models for Incident 2, a left temporoparietal impact.

Two of the models for Incident 2 did produce good reproductions of tangential gunshot wounds, but this was not the mechanism being reconstructed. Post hoc matching of clinical images to synthetic ballistic injury models is suitable for proof of concept, but care is needed in reconstructions to ensure that incorrect conclusions are not drawn where the features produced in models do not match the circumstances of the incident.

Skin wound appearances on models shot wearing a helmet are very different from the same models shot without a helmet.

Positive features of the model include realistic internal fracture lines and the ability to place a helmet to reproduce military scenarios.

Negative features include the post-mortem cut line in the skull (which interferes with fracture propagation in some instances) and the extendable nature of the skin.

Further work is ongoing to address the limitations within the models.
